# De novo familial adenomatous polyposis with germline double heterozygosity of APC/BRCA2: a case report and literature review

**DOI:** 10.1186/s13053-025-00306-x

**Published:** 2025-02-21

**Authors:** Tian-Qi Zhang, Ji-Dong Cai, Cong Li, Yun Xu, Ye Xu

**Affiliations:** 1 Department of Pathology, Fudan University Shanghai Cancer Center, Shanghai, China; 2 Department of Endoscopy, Fudan University Shanghai Cancer Center, Shanghai, China; 3 Department of Colorectal Surgery, Fudan University Shanghai Cancer Center, Shanghai, China; 4 Department of Colorectal Surgery, Fudan University, Shanghai Cancer Center, Dong’an Road, 270, Shanghai, 200032 China

**Keywords:** Familial adenomatous polyposis, APC, BRCA2, Germline double heterozygosity, Colorectal cancer

## Abstract

**Background:**

The widespread application of colonoscopy screening and genetic testing in colorectal cancer (CRC) treatment has led to the identification of a subset of familial adenomatous polyposis (FAP) patients who lack a family history of the disease but harbor germline gene mutations. Moreover, distinct genotypes may be associated with varied clinical presentations and therapeutic options. This case report describes a male patient with de novo FAP who harbored germline double heterozygosity (GDH) for APC and BRCA2 mutations. The patient underwent total colectomy, and genetic testing enabled personalized surveillance and management strategies for his family members.

**Case presentation:**

A 43-year-old male with no family history of cancer presented to the outpatient clinic of the Colorectal Surgery Department with complaints of constipation and hematochezia. Colonoscopy revealed hundreds of polyps throughout the colon and a rectal adenocarcinoma located 5 cm from the anal verge. Gastroduodenal endoscopy did not detect any upper gastrointestinal adenomas. The patient underwent laparoscopic total colectomy with abdominoperineal resection of the rectum and end ileostomy. With the consent of the patient and his family, genetic testing was performed. The index patient was found to carry an APC splicing site mutation (exon 15: c.1744-1G > A) and a BRCA2 missense mutation (exon 17: c.7976G > A: p.R2659K). His daughter was found to have inherited the same germline BRCA2 variant. Additionally, the rectal cancer exhibited proficient DNA mismatch repair (pMMR) status, ERBB2 copy number amplification, and a missense mutation, while the KRAS, NRAS, and BRAF genes were wild-type. Based on the genetic testing results and clinical manifestations, the index patient was diagnosed with familial adenomatous polyposis (FAP) and rectal cancer. Personalized surveillance and management strategies were implemented for the patient and his family, focusing on the risks of extra-colonic diseases and potential malignancies in the prostate, pancreas, breast, and ovaries.

**Conclusion:**

De novo FAP with double germline mutations in APC and BRCA2, along with somatic ERBB2 mutations, is exceptionally rare among hereditary cancer cases. With the rapid advancements in genomics, the detection of multiple gene variants in individuals or families has become increasingly common. Additionally, the application of artificial intelligence (AI) in medical research may provide powerful tools for genetic analysis and clinical decision-making. Consequently, a comprehensive evaluation of family history, a deep understanding of hereditary cancer syndromes, and precise interpretation of genetic mutations are essential for personalized clinical management in the era of precision medicine. However, these tasks pose significant challenges for clinicians and genetic counselors alike.

## Background

FAP is an autosomal dominant inherited disease characterized by hundreds of adenomas in the colon [1]. If left untreated, patients with FAP develop CRC inevitably. Based on the polyp burden, FAP is divided into classical FAP (over 100 adenomas) and attenuated FAP (fewer than 100 adenomas) [2]. Some extracolonic manifestations are associated with FAP, including upper intestinal adenomas, desmoid tumors, hepatoblastoma, thyroid cancer, congenital hypertrophy of the retinal pigment epithelium, brain tumors, and epidermoid cysts [3]. APC, located in 5q21–q22, is the causative gene for FAP [4]. Its mutations lead to truncated proteins, resulting in disruption of the Wnt signaling pathway [5]. Approximately 11–25% of FAP patients without family histories carry de novo APC mutations [6]. However, not all FAP patients have APC mutations, and different APC variants are associated with diverse FAP manifestations [7]. GDH is rare among hereditary cancer syndromes [8], but it has become more frequently detected due to advances in sequencing technology [9]. Interestingly, BRCA1 and BRCA2 are often coinherited in ovarian and breast cancers [10–12]. It is well-known that BRCA1/2 carriers have higher risks of developing breast, ovarian, pancreatic, and prostate cancers, but their effects on CRC are still controversial [13]. On the other hand, ERBB2 mutations or amplifications have been identified in approximately 15–30% of breast cancers and 10–30% of gastric/gastroesophageal cancers and serve as prognostic and predictive biomarkers for those cancers [14]. However, ERBB2-targeted therapies are not currently approved for ERBB2-positive metastatic CRC, which accounts for approximately 7% of all CRC cases [15, 16].

In this study, we describe a case of an index patient with FAP and rectal cancer harboring germline mutations in both APC and BRCA2, as well as somatic ERBB2 mutations. We analyze the genotype-phenotype correlations and propose individualized treatment plans and surveillance strategies for the patient and his family, integrating clinical manifestations, family history, previous studies, and genetic test results.

## Case presentation

**Fig. 1:**
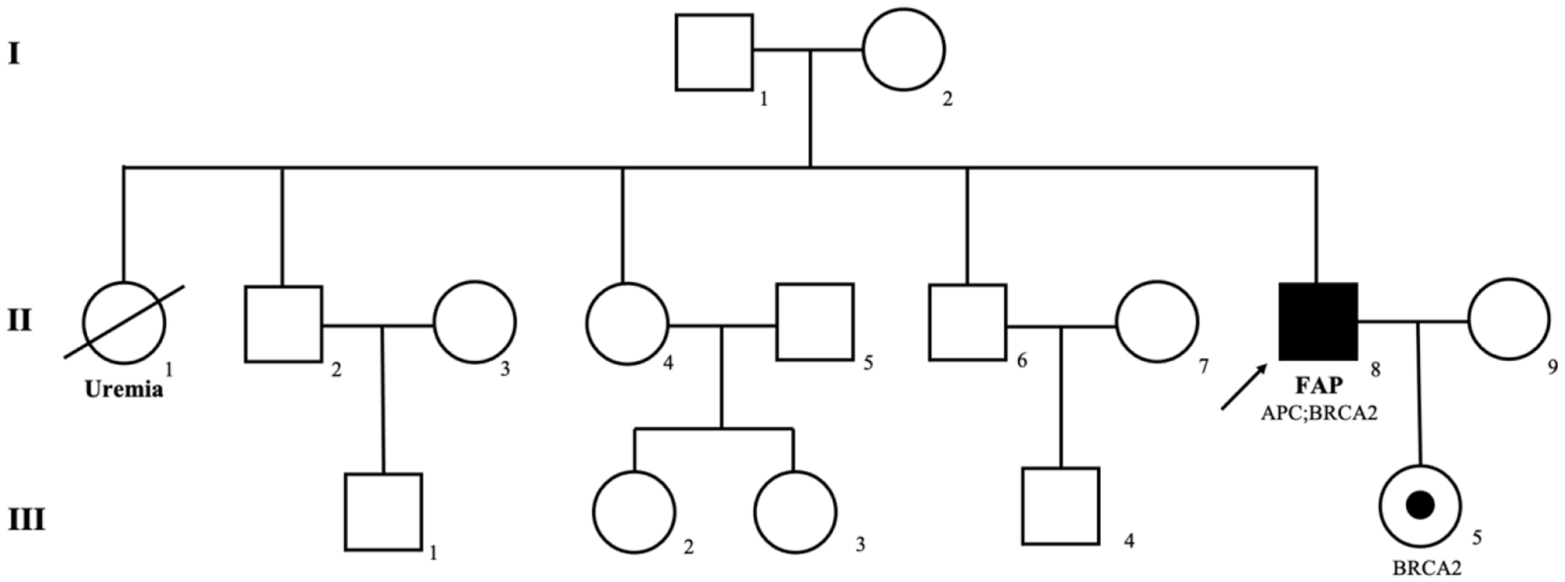
Pedigree of the family. Family members with FAP are shaded. The arrow indicates the proband. Squares and circles are labeled as males and females, respectively. A shaded circle in a blank circle indicates a carrier without disease. Roman numerals mean generations

A 43-year-old man presented to the Colorectal Surgery Department of Fudan University Shanghai Cancer Center (FUSCC) with an eight-year history of constipation and hematochezia. Colonoscopy revealed hundreds of polyps throughout the colon and a rectal adenocarcinoma located 5 cm from the anus. No adenomas were detected in the upper gastrointestinal tract. The patient reported no family history of familial adenomatous polyposis (FAP) or other malignant tumors. Subsequently, laparoscopic total colectomy with abdominoperineal resection of the rectum and end ileostomy was performed. Histopathological examination revealed hundreds of tubular-villous adenomas in the colon, with a moderately differentiated adenocarcinoma (2 × 2 × 1.2 cm) in the rectum. No lymph node metastases or neurovascular invasion were observed. Immunohistochemistry showed pMMR status and HER2 positivity (3+). Given the patient’s FAP-like manifestations, genetic testing was recommended for the patient and his relatives. Results revealed that the index patient carried germline mutations in APC (exon 15: c.1744-1G > A, splice site) and BRCA2 (exon 17: c.G7976A; p.R2659K). His daughter was found to have inherited the BRCA2 mutation. Moreover, somatic mutations in the rectal tumor included ERBB2 copy number amplification and a missense mutation (exon 17: c.G2033A; p.R648Q). No mutations were detected in KRAS, NRAS, or BRAF. The genetic testing results are summarized in Table 1, and the family pedigree is depicted in Fig. 1. Based on the clinical manifestations and genetic testing results, the patient was diagnosed with FAP and rectal cancer. A personalized surveillance plan was developed in collaboration with genetic counselors. First, based on the pathological results, chemotherapy and radiotherapy were not recommended for the index patient. However, follow-up visits every 3 months during the first year were deemed necessary. Second, given the presence of the germline pathogenic BRCA2 variant, the patient was advised to undergo regular screenings for prostate and pancreatic cancers every 6–12 months. Additionally, his daughter was referred to the Obstetrics and Gynecology clinic for consultation regarding surveillance of breast and ovarian cancers, as well as for fertility counseling.

**Table 1:** Somatic and germline mutations detected in the patient

Gene	Variability source	Genotype	Mutation Type	Mutation Site
ERBB2	somatic mutation		copy number amplication	
ERBB2	somatic mutation	heterozygote	missense mutation	exon17: c.G2033A: p.R648Q
APC	germline mutation	heterozygote	slicing site mutation	exon15: c.1744-1G > A
BRCA2	germline mutation	heterozygote	missense mutation	exon17: c.G7976A: p.R2659K

## Discussion

GDH accounts for just 0.2% of all cases of hereditary cancer syndromes, and previous studies have documented co-inheritance of APC with mutations in p53, MSH2, and BRCA1 in FAP families [8, 17–20]. In this case, the index FAP patient carries double pathogenic germline mutations of APC and BRCA2, and his daughter inherited the BRCA2 variant, making this first report in Chinese FAP patients. In addition, ERBB2 somatic mutations were also detected in his rectal cancer samples. Therefore, genotype-phenotype relationships in this patient require further investigation.

### Genotype and phenotype of the APC variant

To date, approximately 3,000 APC variants have been identified in FAP patients [21]. The most common types include nonsense and frameshift mutations, splice site mutations, deep intronic deletions, and missense mutations in a few cases [22, 23]. Exon 15 of the APC gene is a well-established hotspot for germline mutations, particularly at codons 1061 and 1309 [24, 25]. In this case, the FAP patient harbors a splice site mutation in APC (exon 15: c.1744-1G > A), which results from a G to A substitution one nucleotide before the coding exon 15. Most APC pathogenic variants lead to premature termination codons, while aberrant splicing variants, which account for only approximately 6%, result in alternative transcripts [26]. Previous reports have shown that the c.1744G > T mutation causes a premature stop codon, and the c.1744–2 A > G mutation leads to the complete skipping of exon 15 [23, 27]. Similarly, the splice-acceptor site mutation (c.1744-1G > A) at the intron 14–exon 15 boundary causes aberrant splicing, resulting in an abnormal APC protein. This mutation disrupts the β-catenin-binding domain, thereby impairing the Wnt/β-catenin signaling pathway.

Clinically, this specific mutation was first reported in a four-generation Chinese pedigree comprising 45 members, of whom four were affected by classical FAP, including three with CRC [28]. Consistent with our index patient, individuals carrying this mutation developed intestinal symptoms between the ages of 30 and 40 years. Notably, extracolonic manifestations such as diffuse fundic gland polyposis, gastric adenomas, duodenal adenomas, or desmoid tumors were absent in these affected individuals. Moreover, malignant polyps were predominantly located in the rectum. These findings suggest that the APC variant (exon 15: c.1744-1G > A) may be associated with classical FAP with limited extracolonic manifestations. However, further studies are required to validate this genotype-phenotype correlation.

From a clinical management perspective, proctocolectomy is generally recommended for patients with a definitive FAP diagnosis. However, the optimal timing of surgery remains controversial due to its impact on anal function, fertility, and the risk of desmoid tumors [29]. Postponing surgery is often debated, particularly in young, asymptomatic patients who are compliant with surveillance or those with attenuated FAP (AFAP) or a high risk of desmoid tumors [29]. Patients with the APC (c.1744-1G > A) mutation appear to develop symptoms later, exhibit a low risk of desmoid tumors, and carry a higher risk for rectal cancer. Therefore, surgery may be deferred, with close endoscopic monitoring and attention to rectal polyps. This case highlights the potential for APC genotype-guided FAP treatment strategies.

### BCRA2 germline mutation in cancers

The index patient harbors a germline BRCA2 mutation (exon 17: c.G7976A; p.R2659K), characterized by a G to A substitution at the end of exon 17, resulting in an amino acid change from arginine to lysine. This pathogenic mutation impacts mRNA splicing, leading to in-frame skipping of exon 17, loss of a functional domain, and impaired BRCA2 functions, including homologous recombination repair, centrosome regulation, and DNA repair activity. BRCA2 mutations have been associated with increased risks of breast, ovarian, prostate, and pancreatic cancers. Female BRCA2 carriers face a 55% lifetime risk of breast cancer and 10–15% of ovarian cancer, while male carriers have a 7.1% risk by age 70 and 8.4% by age 80 [30–32]. Additionally, BRCA2 mutations are linked to a 40% increased risk of prostate cancer and are detected in 0–17% of pancreatic cancer cases [33, 34]. However, whether BRCA2 mutations increase the risk of colorectal cancer remains controversial; some studies suggest that BRCA1 pathogenic variants may be associated with colorectal cancer risk, while BRCA2 variants are not [35, 36].

Germline mutations in BRCA2 can lead to defects in homologous recombination, which is a critical DNA repair pathway. Tumors with homologous recombination deficiency (HRD) are particularly sensitive to platinum-based chemotherapy and poly ADP-ribose polymerase inhibitors (PARPi) therapy. HRD testing is pivotal for determining BRCA2 involvement in disease progression and provides a rationale for using PARPi as a therapeutic option. While pathogenic BRCA1/2 mutations are major contributors to HRD, additional genomic instability markers—loss of heterozygosity (LOH), telomeric allelic imbalance (TAI), and large-scale state transitions (LST)—can also predict HRD status. Currently, HRD testing relies on next-generation sequencing (NGS), which is time-consuming, costly, tissue-intensive, and has a high failure rate [37]. These challenges have driven the development of AI-based tools that offer more efficient and cost-effective alternatives. Recent studies highlight the transformative potential of AI in HRD detection and cancer research [38]. For example, iPREDICT-HRD predicts HRD from histopathological slides with 99.3% accuracy, demonstrating intratumor heterogeneity through heatmap analyses [37]. MODeepHRD, a multi-omics deep learning framework, integrates transcriptomic, DNA methylation, and mutation data to outperform conventional methods, showing that HRD-positive tumors are significantly associated with improved survival and enhanced responses to platinum-based therapies [39]. Similarly, SigMA enables the detection of HRD mutational signatures from targeted gene panels, bypassing the need for whole-genome sequencing and identifying patients most likely to benefit from PARPi therapy [40]. These AI-enabled tools not only improve the speed and accuracy of HRD testing but also enhance patient stratification and therapeutic decision-making, underscoring their potential to revolutionize precision oncology.

In this case, the index patient carries double germline mutations in APC and BRCA2 but currently presents only with manifestations of FAP. This may be attributed to the higher penetrance of APC mutations and the earlier onset age of FAP compared to cancers associated with BRCA2. A prior report documented coinheritance of APC and BRCA2 mutations in an individual with FAP and intraductal papillary mucinous neoplasm (IPMN), whose siblings also carrying both mutations developed pancreatic cancer [41]. However, a limitation of this case report is the absence of HRD testing to assess the involvement of BRCA2 in the development and progression of colorectal cancer. Such testing would provide valuable insights into the potential use of PARPi in the future management of similar cases.

Consequently, this patient faces an elevated risk of developing male breast cancer, prostate cancer, pancreatic cancer, and other malignancies in the future. We recommend that this patient undergo prostate-specific antigen (PSA) screening, perform regular breast self-examinations, and initiate pancreatic cancer screening at age 50. Additionally, we advise his daughter to undergo regular breast and ovarian cancer screening and consider preimplantation genetic diagnosis (PGD) when planning for pregnancy to prevent the transmission of genetic mutations.

### ERBB2 positivity in CRC

ERBB2-positive CRCs represent a small but distinct subset of cases with unique characteristics. These tumors are more commonly located on the left side of the colon or rectum and have a higher risk of central nervous system metastases. They are frequently associated with the CMS2 molecular subtype and are predominantly found in KRAS/NRAS/BRAF wild-type tumors. ERBB2 amplification often occurs concurrently with ERBB2 mutations, which in turn are frequently accompanied by BRAF and PIK3CA mutations [42]. Despite these findings, no ERBB2-targeted therapies are currently approved for patients with ERBB2-positive mCRC. In this case, sequencing revealed ERBB2 copy number amplification and a missense mutation (exon 17: c.G2033A; p.R648Q), and immunohistochemical analysis demonstrated HER2 positivity (3+) in the rectal cancer specimen. This particular missense mutation has previously been identified in cancers of the bladder, stomach, uterus, prostate, breast, and colon [43, 44]. These results suggest that anti-ERBB2 therapies, such as lapatinib and trastuzumab, could be potential treatment options if metastasis occurs.

To date, the relationship between germline mutations and somatic mutations remains unclear. In this case, germline mutations in APC and BRCA2, as well as somatic mutations in ERBB2, were detected in a single patient. However, the association between ERBB2 mutations and APC or BRCA2 mutations is uncertain. According to a previous study, co-mutation of APC and HER2 in CRC is relatively common [45]. In contrast, the co-occurrence of BRCA2 germline mutations with HER2 amplification or mutation has not been extensively explored in CRC. In breast cancer, HER2 positivity is observed in 2.5% and 3.2% of women with BRCA1 or BRCA2 mutations, respectively, compared to 27.7% and 8.2% for triple-negative tumors [46]. A Chinese study from FUSCC revealed HER2 positivity in 7.3% of BRCA1 germline mutations and 8.7% of BRCA2 germline mutations in breast cancer [47]. Therefore, HER2 positivity is relatively rare in breast cancer patients with BRCA1/2 germline mutations and is likely even less common in CRC. Additionally, the molecular mechanisms underlying this phenotype remain unclear. APC, BRCA2, and ERBB2 are involved in distinct pathways: the Wnt pathway, homologous recombination repair pathway, and epidermal growth factor receptor (EGFR) pathway, respectively. However, potential cross-talk between these pathways has yet to be elucidated and warrants further investigation.

## Conclusion

In conclusion, we present a rare case of a de novo FAP patient with rectal cancer, characterized by double germline mutations in APC and BRCA2, alongside somatic ERBB2 mutations. This case highlights the importance of recognizing that not all FAP patients have a family history. With the rapid advancements in genomic technologies, the identification of multiple gene variants within an individual or family is becoming increasingly common. Furthermore, the integration of AI into medical research is poised to revolutionize the field, offering powerful tools for genetic analysis and clinical decision-making. Therefore, a comprehensive evaluation of family history, a deep understanding of hereditary cancer syndromes, the strategic application of AI tools, and precise interpretation of genetic mutations are critical for tailoring individualized clinical management in the era of precision medicine. However, these advancements also present significant challenges for clinicians and genetic counselors, requiring multidisciplinary expertise and careful consideration of ethical, technical, and clinical implications.

## Data Availability

No datasets were generated or analysed during the current study.

## Abbreviations

FAP: Familial adenomatous polyposis
CRC: Colorectal cancer
GDH: Germline double heterozygosity
FUSCC: Fudan University Shanghai Cancer Center
MMR: Mismatch repair
IPMN: Intraductal papillary mucinous neoplasm
EGFR: Epidermal growth factor receptor
HRD: Homologous recombination deficiency
PARPi: Poly ADP-ribose polymerase inhibitors
AI: Artificial intelligence
PSA: Prostate-specific antigen
PGD: Preimplantation genetic diagnosis
